# *catena*-Poly[[[diaqua­copper(II)]-μ-quinoline-2,3-dicarboxyl­ato-κ^3^ *N*,*O* ^2^:*O* ^3^] monohydrate]

**DOI:** 10.1107/S1600536812043206

**Published:** 2012-10-20

**Authors:** Qiao-Hua Xia, Zhong-Fu Guo, Li Liu, Zhi-kun Wang, Bing Li

**Affiliations:** a College of Sciences, Zhejiang A&F University, Lin’an, Hangzhou, Zhejiang 311300, People’s Republic of China

## Abstract

In the title compound, {[Cu(C_11_H_5_NO_4_)(H_2_O)_2_]·H_2_O}_*n*_, the Cu^II^ ion is five-coordinated by two O atoms and one N atom of two symmetry-related quinoline-2,3-dicarboxyl­ate ligands, and two water mol­ecules. The water mol­ecules occupy basal and apical positions of the square-pyramidal coordination polyhedron. Each quinoline-2,3-dicarboxyl­ate dianion bridges two adjacent Cu^II^ ions, forming a polymeric chain along [010]. The chains are further connected *via* O—H⋯O hydrogen-bonding inter­actions and quinoline ring π–π inter­actions [centroid–centroid distance = 3.725 (4) Å], generating a three-dimensional structure. Lattice water mol­ecules participate in the crystal structure *via* O—H⋯O hydrogen bonds.

## Related literature

For background to complexes based on quinoline-2,3-dicarboxylic acid, see: Li & Liu (2010 ▶).
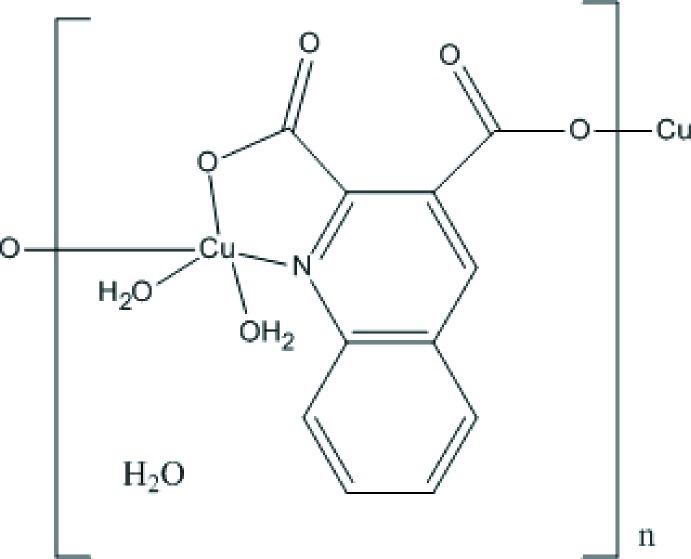


## Experimental

### 

#### Crystal data

[Cu(C_11_H_5_NO_4_)(H_2_O)_2_]·H_2_O
*M* *_r_* = 332.76
Triclinic, 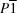

*a* = 7.0284 (14) Å
*b* = 7.5836 (15) Å
*c* = 13.276 (3) Å
α = 104.74 (3)°
β = 91.19 (3)°
γ = 116.03 (3)°
*V* = 607.7 (2) Å^3^
*Z* = 2
Mo *K*α radiation
μ = 1.83 mm^−1^
*T* = 293 K
0.43 × 0.34 × 0.20 mm

#### Data collection

Rigaku R-AXIS RAPID diffractometer
Absorption correction: multi-scan (*ABSCOR*; Higashi, 1995 ▶) *T*_min_ = 0.763, *T*_max_ = 0.854
5813 measured reflections
2696 independent reflections
2382 reflections with *I* > 2σ(*I*)
*R*_int_ = 0.048

#### Refinement

*R*[*F* ^2^ > 2σ(*F* ^2^)] = 0.037
*wR*(*F* ^2^) = 0.104
*S* = 1.10
2696 reflections
199 parameters
10 restraints
H atoms treated by a mixture of independent and constrained refinement
Δρ_max_ = 0.73 e Å^−3^
Δρ_min_ = −0.74 e Å^−3^

### 

Data collection: *RAPID-AUTO* (Rigaku, 1998 ▶); cell refinement: *RAPID-AUTO*; data reduction: *CrystalStructure* (Rigaku/MSC, 2004 ▶); program(s) used to solve structure: *SHELXS97* (Sheldrick, 2008 ▶); program(s) used to refine structure: *SHELXL97* (Sheldrick, 2008 ▶); molecular graphics: *SHELXTL* (Sheldrick, 2008 ▶); software used to prepare material for publication: *SHELXL97*.

## Supplementary Material

Crystal structure: contains datablock(s) global, I. DOI: 10.1107/S1600536812043206/bh2454sup1.cif

Structure factors: contains datablock(s) I. DOI: 10.1107/S1600536812043206/bh2454Isup2.hkl

Additional supplementary materials:  crystallographic information; 3D view; checkCIF report

## Figures and Tables

**Table 1:** Hydrogen-bond geometry (Å, °)

*D*—H⋯*A*	*D*—H	H⋯*A*	*D*⋯*A*	*D*—H⋯*A*
O*W*1—H1⋯O*W*3^i^	0.81 (2)	2.11 (2)	2.916 (4)	177 (4)
O*W*1—H2⋯O3^i^	0.81 (2)	2.15 (2)	2.944 (3)	166 (5)
O*W*2—H3⋯O1^ii^	0.87 (2)	2.12 (2)	2.962 (3)	163 (4)
O*W*2—H4⋯O2^iii^	0.89 (2)	2.29 (2)	3.174 (3)	178 (4)
O*W*3—H5⋯O1^iv^	0.82 (2)	1.96 (2)	2.775 (4)	170 (5)
O*W*3—H6⋯O3^ii^	0.80 (2)	2.12 (2)	2.909 (3)	169 (5)

Symmetry codes: (i) 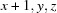; (ii) 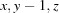; (iii) 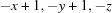; (iv) 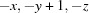.

**Fig. 1.**
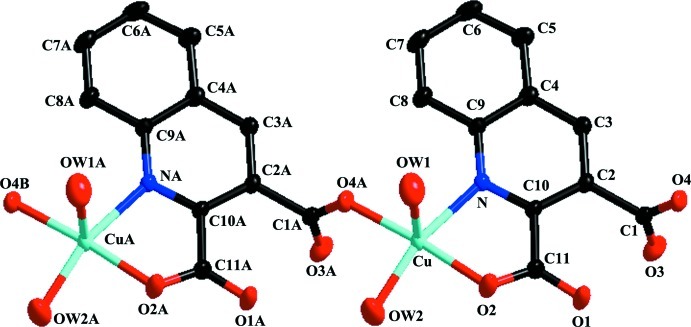
The molecular structure of the title complex, with 50% probability displacement ellipsoids for non-H atoms. Symmetry codes: (A) x, -1 + y, z; (B) x, -2 + y, z.

**Fig. 2.**
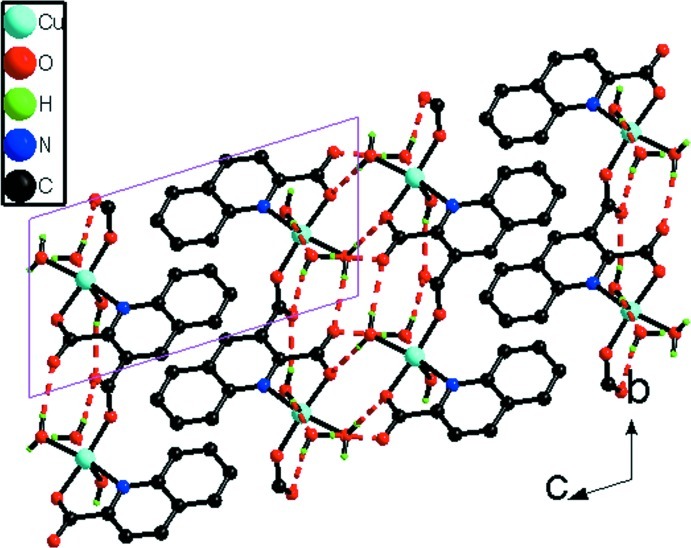
Crystal packing diagram for the title compound. All atoms are shown as isotropic spheres of arbitrary size. H atoms bonded to C atoms are omitted for clarity. The H-bonding interactions are shown as red dashed lines.

## supplementary crystallographic information

### Comment

The asymmetric unit of the title complex contains one Cu^II^ ion, one quinoline-2,3-dicarboxylate dianion, two coordinated water molecules and one lattice water molecule (Fig. 1). The Cu^II^ ion is five-coordinated within a square-pyramidal [CuNO_4_]coordination geometry. Five coordination arises from two O atoms and one N atom belonging to two 2,3-quinolinedicarboxylate ligands (Li & Liu, 2010), and two water molecules. The Cu—O bond lengths vary from 1.9403 (19) to 2.320 (3) Å, and the Cu—N distance is 2.096 (2) Å. Each Cu^II^ ion interacts with adjacent Cu^II^*via* the bridging mode of the dianion, forming a one-dimensional framework. The resulting chains are further connected through O—H···O hydrogen bonding interactions between the O atoms of quinoline-2,3-dicarboxylate dianion, coordinated water molecules and one lattice water molecule [O···O separations in the range 2.775 (4)–3.174 (3) Å]. Additionally, π–π [3.725 (4) Å] interactions between quinoline rings are involved in the formation of the three-dimensional supramolecular structure (Fig. 2). The shortest Cu···Cu separation along the polymeric chain is 7.5836 (2) Å.

### Experimental

All commercially obtained reagent grade chemicals were used without further purification. A mixture of copper chloride dihydrate (0.1708 g, 1 mmol) and 2,3-quinolinedicarboxylic acid (0.2171 g, 1 mmol) was added into 20 ml of water with few drops of ammonia solution, and then stirred for 1 h. After 2 days, blue crystals of the title complex were collected by filtration, washed with distilled water, and dried in air.

### Refinement

All H atoms bonded to C atoms were positioned geometrically and refined using the riding model with C—H = 0.93 Å. The H atoms of water molecules were located from a difference map and were restrained at distances O—H = 0.83 (1) Å. The separation between H atoms in the same water molecule was restrained to H···H = 1.35 (1) Å. Cu and OW2 atoms were restrained to have similar displacement parameters (*SIMU* restraint; Sheldrick, 2008). Isotropic displacement parameters for H atoms were calculated as *U*_iso_(H) = 1.2*U*_eq_(carrier C) and *U*_iso_(H) = 1.5*U*_eq_(carrier O).

### Crystal data

**Table:**

[Cu(C_11_H_5_NO_4_)(H_2_O)_2_]·H_2_O	*Z* = 2
*M**_r_* = 332.76	*F*(000) = 338
Triclinic, *P*1	*D*_x_ = 1.818 Mg m^−^^3^
Hall symbol: -P 1	Mo *K*α radiation, λ = 0.71073 Å
*a* = 7.0284 (14) Å	Cell parameters from 5355 reflections
*b* = 7.5836 (15) Å	θ = 3.1–27.5°
*c* = 13.276 (3) Å	µ = 1.83 mm^−^^1^
α = 104.74 (3)°	*T* = 293 K
β = 91.19 (3)°	Block, blue
γ = 116.03 (3)°	0.43 × 0.34 × 0.20 mm
*V* = 607.7 (2) Å^3^	

### Data collection

**Table:**

Rigaku R-AXIS RAPID diffractometer	2696 independent reflections
Radiation source: fine-focus sealed tube	2382 reflections with *I* > 2σ(*I*)
Graphite monochromator	*R*_int_ = 0.048
ω scans	θ_max_ = 27.5°, θ_min_ = 3.1°
Absorption correction: multi-scan (*ABSCOR*; Higashi, 1995)	*h* = −8→9
*T*_min_ = 0.763, *T*_max_ = 0.854	*k* = −9→8
5813 measured reflections	*l* = −17→17

### Refinement

**Table:**

Refinement on *F*^2^	Primary atom site location: structure-invariant direct methods
Least-squares matrix: full	Secondary atom site location: difference Fourier map
*R*[*F*^2^ > 2σ(*F*^2^)] = 0.037	Hydrogen site location: inferred from neighbouring sites
*wR*(*F*^2^) = 0.104	H atoms treated by a mixture of independent and constrained refinement
*S* = 1.10	*w* = 1/[σ^2^(*F*_o_^2^) + (0.0501*P*)^2^ + 0.4308*P*] where *P* = (*F*_o_^2^ + 2*F*_c_^2^)/3
2696 reflections	(Δ/σ)_max_ < 0.001
199 parameters	Δρ_max_ = 0.73 e Å^−^^3^
10 restraints	Δρ_min_ = −0.74 e Å^−^^3^
0 constraints	

### Fractional atomic coordinates and isotropic or equivalent isotropic displacement parameters (Å^2^)

**Table:**

	*x*	*y*	*z*	*U*_iso_*/*U*_eq_	
Cu	0.30340 (5)	0.43833 (4)	0.17117 (2)	0.02367 (13)	
O1	0.2017 (5)	0.8429 (3)	0.07483 (18)	0.0454 (6)	
O2	0.3062 (4)	0.6111 (3)	0.08503 (16)	0.0323 (5)	
O3	−0.0584 (3)	1.0076 (3)	0.20096 (18)	0.0351 (5)	
O4	0.2863 (3)	1.2456 (3)	0.24768 (16)	0.0279 (4)	
OW1	0.6719 (4)	0.5956 (4)	0.2208 (2)	0.0448 (6)	
H1	0.723 (7)	0.518 (5)	0.198 (4)	0.067*	
H2	0.733 (7)	0.701 (4)	0.205 (4)	0.067*	
OW2	0.2962 (5)	0.2398 (4)	0.0373 (2)	0.0465 (6)	
H3	0.292 (7)	0.126 (5)	0.042 (4)	0.070*	
H4	0.406 (6)	0.284 (6)	0.003 (4)	0.070*	
OW3	−0.1618 (5)	0.3035 (4)	0.1425 (2)	0.0516 (7)	
H5	−0.185 (8)	0.265 (7)	0.0779 (15)	0.077*	
H6	−0.140 (8)	0.226 (6)	0.166 (3)	0.077*	
N	0.2639 (3)	0.6617 (3)	0.28625 (17)	0.0193 (4)	
C1	0.1293 (4)	1.0719 (4)	0.2397 (2)	0.0229 (5)	
C2	0.1836 (4)	0.9435 (4)	0.2947 (2)	0.0195 (5)	
C3	0.1917 (4)	0.9836 (4)	0.4014 (2)	0.0216 (5)	
H3A	0.1706	1.0930	0.4396	0.026*	
C4	0.2314 (4)	0.8615 (4)	0.4539 (2)	0.0208 (5)	
C5	0.2409 (4)	0.8966 (4)	0.5644 (2)	0.0259 (6)	
H5A	0.2254	1.0074	0.6055	0.031*	
C6	0.2726 (5)	0.7685 (5)	0.6109 (2)	0.0306 (6)	
H6A	0.2771	0.7918	0.6833	0.037*	
C7	0.2985 (5)	0.6019 (5)	0.5502 (2)	0.0312 (6)	
H7A	0.3172	0.5143	0.5827	0.037*	
C8	0.2966 (5)	0.5668 (4)	0.4442 (2)	0.0277 (6)	
H8A	0.3183	0.4581	0.4056	0.033*	
C9	0.2620 (4)	0.6941 (4)	0.3922 (2)	0.0200 (5)	
C10	0.2271 (4)	0.7826 (4)	0.2399 (2)	0.0201 (5)	
C11	0.2456 (5)	0.7447 (4)	0.1240 (2)	0.0255 (6)	

### Atomic displacement parameters (Å^2^)

**Table:**

	*U*^11^	*U*^22^	*U*^33^	*U*^12^	*U*^13^	*U*^23^
Cu	0.0347 (2)	0.02115 (19)	0.0221 (2)	0.01646 (16)	0.00703 (14)	0.01064 (13)
O1	0.095 (2)	0.0415 (12)	0.0239 (12)	0.0488 (14)	0.0131 (12)	0.0165 (9)
O2	0.0555 (13)	0.0351 (11)	0.0250 (11)	0.0322 (11)	0.0176 (10)	0.0170 (9)
O3	0.0349 (11)	0.0418 (12)	0.0381 (13)	0.0222 (10)	0.0005 (9)	0.0186 (10)
O4	0.0396 (11)	0.0189 (9)	0.0266 (11)	0.0122 (9)	0.0022 (8)	0.0112 (8)
OW1	0.0312 (12)	0.0355 (12)	0.0668 (19)	0.0131 (11)	0.0090 (11)	0.0173 (12)
OW2	0.0751 (18)	0.0352 (12)	0.0353 (12)	0.0300 (13)	0.0176 (12)	0.0108 (8)
OW3	0.084 (2)	0.0524 (15)	0.0269 (13)	0.0385 (15)	0.0068 (13)	0.0121 (11)
N	0.0243 (10)	0.0177 (10)	0.0182 (11)	0.0109 (9)	0.0034 (8)	0.0071 (8)
C1	0.0340 (14)	0.0258 (13)	0.0168 (13)	0.0200 (12)	0.0052 (10)	0.0071 (10)
C2	0.0198 (11)	0.0191 (11)	0.0223 (13)	0.0092 (10)	0.0029 (9)	0.0099 (9)
C3	0.0235 (12)	0.0233 (12)	0.0217 (14)	0.0138 (11)	0.0042 (10)	0.0072 (10)
C4	0.0171 (11)	0.0252 (12)	0.0224 (14)	0.0100 (10)	0.0044 (9)	0.0100 (10)
C5	0.0257 (13)	0.0348 (14)	0.0221 (14)	0.0170 (12)	0.0064 (10)	0.0103 (11)
C6	0.0302 (14)	0.0482 (17)	0.0208 (14)	0.0203 (14)	0.0078 (11)	0.0176 (12)
C7	0.0339 (15)	0.0401 (16)	0.0292 (16)	0.0198 (14)	0.0044 (12)	0.0202 (13)
C8	0.0349 (14)	0.0287 (13)	0.0258 (15)	0.0176 (13)	0.0025 (11)	0.0128 (11)
C9	0.0213 (11)	0.0208 (11)	0.0198 (13)	0.0098 (10)	0.0024 (9)	0.0089 (9)
C10	0.0249 (12)	0.0202 (11)	0.0182 (13)	0.0111 (10)	0.0026 (9)	0.0090 (9)
C11	0.0378 (15)	0.0224 (12)	0.0197 (14)	0.0154 (12)	0.0053 (11)	0.0088 (10)

### Geometric parameters (Å, º)

**Table:**

Cu—O2	1.9403 (19)	C5—C6	1.365 (4)
Cu—O4^i^	1.9463 (19)	C5—H5A	0.9300
Cu—OW2	1.999 (3)	C11—C10	1.511 (4)
Cu—N	2.096 (2)	C7—C8	1.362 (4)
Cu—OW1	2.320 (3)	C7—C6	1.403 (4)
O2—C11	1.264 (3)	C7—H7A	0.9300
OW2—H3	0.872 (18)	C10—C2	1.413 (4)
OW2—H4	0.888 (18)	C6—H6A	0.9300
N—C10	1.332 (3)	C8—H8A	0.9300
N—C9	1.367 (3)	OW3—H5	0.820 (19)
OW1—H1	0.813 (18)	OW3—H6	0.803 (18)
OW1—H2	0.810 (18)	C2—C3	1.365 (4)
O1—C11	1.231 (3)	C2—C1	1.517 (3)
C9—C8	1.416 (3)	C3—H3A	0.9300
C9—C4	1.430 (4)	C1—O3	1.234 (4)
C4—C3	1.403 (4)	C1—O4	1.270 (3)
C4—C5	1.418 (4)	O4—Cu^ii^	1.9463 (19)
			
O2—Cu—O4^i^	175.29 (9)	C4—C5—H5A	119.8
O2—Cu—OW2	86.24 (10)	O1—C11—O2	125.0 (3)
O4^i^—Cu—OW2	89.79 (10)	O1—C11—C10	119.1 (2)
O2—Cu—N	81.69 (8)	O2—C11—C10	115.9 (2)
O4^i^—Cu—N	101.87 (8)	C8—C7—C6	120.8 (3)
OW2—Cu—N	165.40 (10)	C8—C7—H7A	119.6
O2—Cu—OW1	95.74 (10)	C6—C7—H7A	119.6
O4^i^—Cu—OW1	87.18 (9)	N—C10—C2	123.3 (2)
OW2—Cu—OW1	95.84 (12)	N—C10—C11	115.7 (2)
N—Cu—OW1	93.51 (10)	C2—C10—C11	120.9 (2)
C11—O2—Cu	116.02 (17)	C5—C6—C7	120.5 (3)
Cu—OW2—H3	117 (3)	C5—C6—H6A	119.7
Cu—OW2—H4	116 (3)	C7—C6—H6A	119.7
H3—OW2—H4	101 (2)	C7—C8—C9	120.8 (3)
C10—N—C9	119.2 (2)	C7—C8—H8A	119.6
C10—N—Cu	108.84 (17)	C9—C8—H8A	119.6
C9—N—Cu	131.95 (17)	H5—OW3—H6	112 (3)
Cu—OW1—H1	112 (3)	C3—C2—C10	118.0 (2)
Cu—OW1—H2	113 (3)	C3—C2—C1	119.3 (2)
H1—OW1—H2	111 (3)	C10—C2—C1	122.7 (2)
N—C9—C8	120.9 (2)	C2—C3—C4	120.7 (2)
N—C9—C4	120.7 (2)	C2—C3—H3A	119.6
C8—C9—C4	118.4 (2)	C4—C3—H3A	119.6
C3—C4—C5	122.9 (2)	O3—C1—O4	127.4 (2)
C3—C4—C9	118.0 (2)	O3—C1—C2	118.7 (2)
C5—C4—C9	119.1 (2)	O4—C1—C2	113.6 (2)
C6—C5—C4	120.3 (3)	C1—O4—Cu^ii^	127.31 (19)
C6—C5—H5A	119.8		

Symmetry codes: (i) *x*, *y*−1, *z*; (ii) *x*, *y*+1, *z*.

### Hydrogen-bond geometry (Å, º)

**Table:**

*D*—H···*A*	*D*—H	H···*A*	*D*···*A*	*D*—H···*A*
O*W*1—H1···O*W*3^iii^	0.81 (2)	2.11 (2)	2.916 (4)	177 (4)
O*W*1—H2···O3^iii^	0.81 (2)	2.15 (2)	2.944 (3)	166 (5)
O*W*2—H3···O1^i^	0.87 (2)	2.12 (2)	2.962 (3)	163 (4)
O*W*2—H4···O2^iv^	0.89 (2)	2.29 (2)	3.174 (3)	178 (4)
O*W*3—H5···O1^v^	0.82 (2)	1.96 (2)	2.775 (4)	170 (5)
O*W*3—H6···O3^i^	0.80 (2)	2.12 (2)	2.909 (3)	169 (5)

Symmetry codes: (i) *x*, *y*−1, *z*; (iii) *x*+1, *y*, *z*; (iv) −*x*+1, −*y*+1, −*z*; (v) −*x*, −*y*+1, −*z*.
